# Spontaneous Thrombosis of the Aortic Arch after Outpatient Urologic Procedure

**DOI:** 10.5811/cpcem.2018.7.38796

**Published:** 2018-08-15

**Authors:** Dean Kerenick, Josh Clore, Julian Jakubowski

**Affiliations:** Marietta Memorial Health System, Department of Emergency Medicine, Marietta, Ohio

## Abstract

A healthy, 42-year-old woman presented to a local community hospital with abdominal pain and left arm pain after laser stone ablation and ureteral stenting performed earlier that day. She was diagnosed with a spontaneous aortic thrombus and embolization of the radial, ulnar and splenic arteries and transferred to a tertiary care facility for cardiothoracic surgery evaluation. This case report discusses her emergency department course, disposition, and one-year outcome.

## INTRODUCTION

Spontaneous arterial occlusion is an uncommon but critical clinical entity with significant potential for morbidity and mortality. Stroke, infarct of solid organs such as kidney or spleen, mesenteric ischemia, ischemic colitis, renal failure, myocardial infarction, and loss of limb are several sequelae that require prompt diagnosis and intervention in the emergency department (ED).^1^ Clinical presentations range from straightforward, such as a painful, pulseless extremity, to more complex and nuanced cases such as abdominal pain due to organ ischemia that can mimic renal colic, colitis, or mechanical back pain.^2^ Diagnostic modalities used to confirm arterial occlusion include nonspecific laboratory analysis such as serum lactate acid level, sonographic imaging, computed tomography (CT), and arteriography and digital subtraction arteriography. The mainstay of treatment is anticoagulation with heparin.^1^–^3^ Other possible treatment includes surgical embolectomy/thrombectomy, clot dissolution with tissue plasminogen activator via systemic administration, or catheter-directed thrombolysis.^3^–^5^ Secondary treatment goals include pain management and searching for the underlying cause and sequelae of thrombosis.^1^–^3^

## CASE REPORT

A 42-year-old female presented to the ED of a community hospital six hours after lithotripsy and laser stone ablation for left ureterolithiasis with complaints of sudden onset of non-radiating, left-sided, aching abdominal pain for one hour. It was associated with mild nausea and left arm pain that she described as an aching, throbbing sensation. She rated her discomfort as severe in intensity. The pain was unchanged after taking a hydrocodone/acetaminophen 5/325 milligrams (mg) tablet at onset of symptoms. She denied any fever, chills, shortness of breath, or chest pain. She appeared quite uncomfortable.

Past medical history was significant only for kidney stones. She denied any family history of blood-clotting disorders. She had a past surgical history of cesarean section, and recent stone ablation and ureteral stenting described above. The only medication she took was hydrocodone 5/325mg tablets, prescribed post-operatively. She denied any smoking or illicit drug use. The patient admitted to an occasional alcoholic beverage.

Physical exam revealed an overweight female in obvious discomfort with an oral temperature of 97.6°F, heart rate of 92 beats per minute, respirations of 22 breaths per minute, and a blood pressure of 93/60 millimeters of mercury. Significant physical findings included pallor of the left distal forearm with no palpable radial or ulnar pulses, and slightly delayed capillary refill to the fingers of the left hand. Right radial and bilateral pedal pulses were 2+. The abdomen was soft and mildly tender in the left middle and lower quadrants with diminished bowel sounds. There was some mild, left costovertebral angle tenderness.

A working differential of arterial occlusion of the left arm, sepsis secondary to urinary tract infection or pyelonephritis, local peritonitis from ureteral rupture, mesenteric ischemia, and abdominal organ injury from lithotripsy was used to formulate the initial work-up. Vascular surgery was consulted and agreed to see the patient emergently in the ED. Diagnostic studies included a complete blood count (CBC), comprehensive metabolic panel, lactic acid, blood cultures, computed tomography angiography (CTA) of the left upper extremity and CT of abdomen and pelvis, pain and nausea control with doses of fentanyl and ondansetron.

**Image 1:**
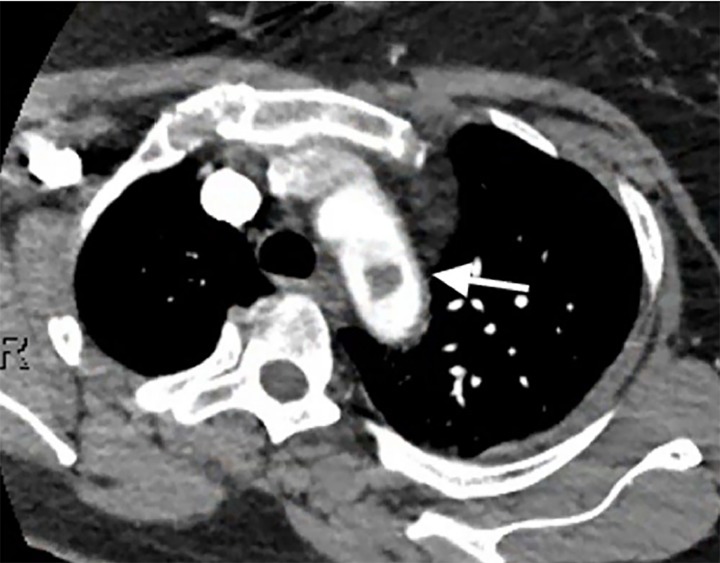
Computed tomography showing thrombus in the aortic arch (arrow).

**Image 2:**
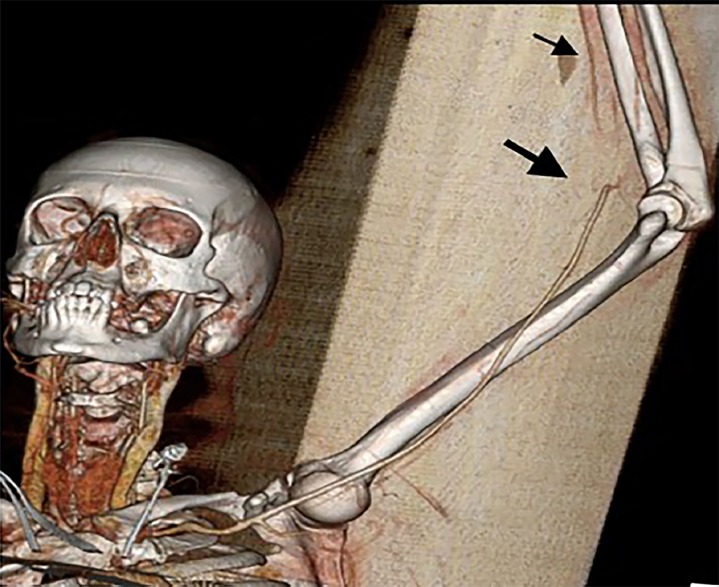
Three-dimensional reconstruction of computed arteriography of forearm showing occlusion of the brachial artery (wide arrow) with reconstitution distally (thin arrow).

**Image 3:**
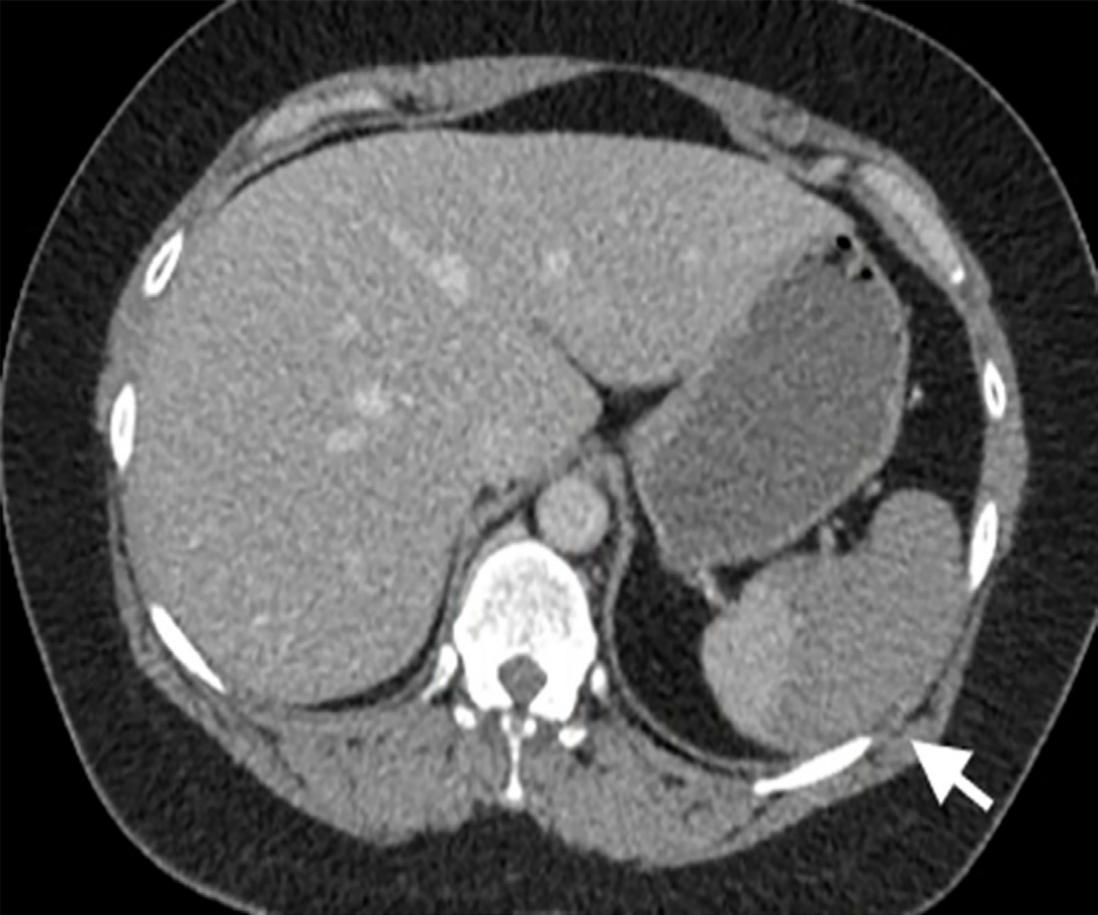
Computed tomography of the abdomen showing decreased contrast uptake in the spleen consistent with large area of infarct (arrow).

CBC showed a white blood count of 16.2 and 96% neutrophils, a hemoglobin level of 11.3, and platelets of 218. Chemistries were within normal limits, except for a mild hypokalemia at 3.3 (repleted intravenously) and a lactic acid elevated at 4.0. CTA of the left upper extremity revealed a non-occlusive thrombus in the aortic arch extending into the origin of the left subclavian artery measuring 12 millimeters (mm) by 12 mm (Image 1), as well as thromboembolic occlusion of the distal left brachial artery at the elbow with reconstitution at the level of the proximal radial and ulnar arteries (Image 2). CT of the abdomen and pelvis showed majority of the spleen was non-enhancing, suggesting a large infarct (Image 3). Given initial concern for sepsis, broad-spectrum coverage with vancomycin and piperacillin/tazobactam was initiated. Vascular surgery recommended transfer to a tertiary facility for possible urgent vs. emergent aortic thrombectomy as our facility did not have cardiovascular surgical capabilities. The patient was heparinized in the interim. Initial coagulation studies, obtained after initial heparin bolus, showed international normalized ratio range (INR) of 1.25 and partial thromboplastin time of 74. Consultation with the cardiovascular surgeon was obtained at the nearest tertiary facility. They agreed with the possible need for urgent intervention, and the patient was accepted for transfer with no further recommendations regarding her care at that time. The patient’s pain was initially treated with intermittent doses of opioids. While awaiting transport, she became increasingly restless and unable to remain still in bed causing dislodgement of monitoring equipment and intravenous access. Due to the intractable pain, compliance with care, and concern for patient safety during transport, the decision was made to intubate her. Routine rapid sequence intubation was performed. Sedation and analgesia with propofol and fentanyl was initiated. The patient was adequately sedated at this point and stable. Transport to the tertiary facility proceeded without further issue.

**CPC-EM Capsule**

What do we already know about this clinical entity?

Aortic thrombus is rare with only a handful of cases noted in the literature. Almost all occurrences are diagnosed after presentation as secondary downstream embolization.

What makes this presentation of disease reportable?

Rare occurrence coupled with life-and-limb threat make this a no-miss diagnosis in the emergency department.

What is the major learning point?

Prompt diagnosis and cardiovascular surgery referral is imperative. Pain from organ ischemia will require aggressive care with possible intubation and sedation.

How might this improve emergency medicine practice?

Keeping the differential of aortic thrombus in mind with any arterial occlusion can expedite appropriate referral and coordination of care.

At the tertiary facility, the patient remained stable. She experienced return of circulation to the upper extremity without intervention. A repeat CTA of the aorta several hours after arrival showed resolution of the aortic thrombus. The remainder of the patient’s hospital stay was uneventful. No underlying pathology was identified as the cause of her acute thrombus formation. She was transitioned to warfarin and discharged home to follow up with her primary care physician for management of her anticoagulation. Several weeks after this event, the patient had outpatient workup for left chest discomfort. INR at that time was therapeutic, and a CTA of the chest showed no evidence of pulmonary embolism. There was a reactive left lower lobe process with a small pleural effusion, believed to be related to a perisplenic fluid collection. The spleen was noted to be slightly larger than on prior study and continued to show changes consistent with an infarct. Her symptoms resolved with supportive care, and follow-up studies showed resolution of the inflammatory process and subsequent splenic atrophy. She currently remains on warfarin and has experienced no post-embolic sequelae other than splenic atrophy.

## DISCUSSION

Thrombus in the aortic arch is an infrequent clinical event with few case reports in the recent literature. In a case series looking at thoracic aortic thrombosis, nine cases were found over a five-year period with only two involving the aortic arch.^6^ Generally, a thrombus is formed secondary to an atherosclerotic plaque with other potential causes including coagulation disorders, paraneoplastic process, cardiac structural abnormalities, or hypercoagulable states.^1^,^3^ In all cases reported in our literature review, the diagnosis was made after an acute presentation for an ischemic limb.

Treatment includes both medical and surgical options. The most common approach is systemic anticoagulation with heparin and transition to oral warfarin or thrombolysis and anticoagulation. While this is effective in most cases, re-embolization is a concern.^1^–^3^ Until recent technological advancements, surgical treatment required cardiopulmonary bypass and carried a high morbidity with up to 14% of patients suffering cerebral insult.^7^ More recently, endovascular therapy has been used in a few cases with good results and avoids postoperative morbidity.^8^–^10^ No studies were found that compared endovascular vs. open surgical embolectomy and repair. In cases and investigations reviewed, all patients were discharged home on some form of anticoagulation, with warfarin the most common agent. No consensus regarding standard treatment was described in the literature. ED treatment remains systemic anticoagulation on heparin and supportive care.^2^

## CONCLUSION

Aortic thrombus and subsequent downstream embolization presents a time-sensitive and life-threatening challenge to the emergency physician. Rapid identification of aortic involvement and subsequent cardiothoracic surgical evaluation is critical. Disposition at a tertiary center with the availability of both endovascular and open cardiopulmonary bypass capabilities is necessary. The physician should be prepared to administer thrombolytic therapy to the hemodynamically unstable patient, as well as deeply sedate the patient if pain cannot otherwise be controlled.

Documented patient informed consent and/or Institutional Review Board approval has been obtained and filed for publication of this case report.
